# Tracing the history of LINE and SINE extinction in sigmodontine rodents

**DOI:** 10.1186/s13100-019-0164-5

**Published:** 2019-05-21

**Authors:** Lei Yang, LuAnn Scott, Holly A. Wichman

**Affiliations:** 1 Department of Biological Sciences, University of Idaho, Moscow, ID USA; 2 Institute for Bioinformatics and Evolutionary Studies, University of Idaho, Moscow, ID USA

## Abstract

**Background:**

L1 retrotransposons have co-evolved with their mammalian hosts for the entire history of mammals and currently compose ~ 20% of a mammalian genome. B1 retrotransposons are dependent on L1 for retrotransposition and span the evolutionary history of rodents since their radiation. L1s were found to have lost their activity in a group of South American rodents, the Sigmodontinae, and B1 inactivation preceded the extinction of L1 in the same group. Consequently, a basal group of sigmodontines have active L1s but inactive B1s and a derived clade have both inactive L1s and B1s. It has been suggested that B1s became extinct during a long period of L1 quiescence and that L1s subsequently reemerged in the basal group.

**Results:**

Here we investigate the evolutionary histories of L1 and B1 in the sigmodontine rodents and show that L1 activity continued until after the L1-extinct clade and the basal group diverged. After the split, L1 had a small burst of activity in the former group, followed by extinction. In the basal group, activity was initially low but was followed by a dramatic increase in L1 activity. We found the last wave of B1 retrotransposition was large and probably preceded the split between the two rodent clades.

**Conclusions:**

Given that L1s had been steadily retrotransposing during the time corresponding to B1 extinction and that the burst of B1 activity preceding B1 extinction was large, we conclude that B1 extinction was not a result of L1 quiescence. Rather, the burst of B1 activity may have contributed to L1 extinction both by competition with L1 and by putting strong selective pressure on the host to control retrotransposition.

**Electronic supplementary material:**

The online version of this article (10.1186/s13100-019-0164-5) contains supplementary material, which is available to authorized users.

## Background

LINEs (Long INterspersed Elements) are autonomous non-LTR retrotransposons. They move through an RNA intermediate, but have non-homologous ends and use target-primed reverse transcription [1]. L1 (LINE-1) is the most successful family of LINEs in eutherian mammals [2] and comprise ~ 20% of a mammalian genome [3–7]. A functional full-length L1 is typically 6000–7000 bp long and composed of a 5′ untranslated region (5’UTR) harboring an RNA polymerase II promoter, two non-overlapping open reading frames (ORFs) known as ORF1 and ORF2 and a 3’UTR followed by a poly-adenosine sequence [8]. The structure of L1 can be diverse among different mammals, particularly in the 5’UTR and ORF1 [5]. The ORF-encoded proteins are strictly required for L1 retrotransposition and are highly *cis*-preferential [9, 10]. L1s are adenosine rich (~ 40%) on their coding strand, which results in biased codon usage compared to host genes [11, 12], elongation defects [13], and premature RNA splicing [14]. This A-richness contributes to the inefficiency of L1 retrotransposition and is proposed to regulate the genes in their vicinity [13].

SINEs (Short INterspersed Elements) are relatively short, non-autonomous, non-LTR retrotransposons. SINEs do not encode proteins for their own retrotransposition and depend on the reverse transcriptase encoded by other transposable elements such as LINEs [15, 16]. Although L1s are highly *cis*-preferential [9, 10], SINEs can take advantage of L1-encoded proteins for their own retrotransposition [15–17], and L1 ORF2 protein is sufficient to drive B1 retrotransposition. Despite their short length, SINEs account for ~ 10% of a typical mammalian genome due to their high copy numbers [3, 4]. Among the ~ 70 SINE families found in mammals [18], B1 is the most abundant in mouse [4] and possibly most rodent species [19]. B1s are derived from the RNA component of signal recognition particle 7SL RNA [20, 21] and share features with its ancestors. A functional B1 is ~ 150 bp long and transcribed by RNA polymerase III with the aid of its two transcription factor binding boxes [22, 23]. B1 sequences are rich in CpG sites, which are methylated and thus prone to mutation in mammalian genomes [24], and the elevated mutation rate is pronounced compared to the A-rich L1s [25, 26].

Both L1 and B1 have long histories of co-evolution with their host genomes. Unlike some LTR retrotransposons [27, 28], there is no known targeted mechanism for L1 excision and thus L1s persist in the genome unless they are removed by non-specific mechanisms. L1s can be found in all placental mammals and marsupials [2, 5, 29]. Mammalian L1s evolve as master lineages so that a single or a few lineages are responsible for the total retrotransposition in a short time window [30–33]. New master elements replace the old ones, eventually dominating retrotransposition, and this replacement process happens recurrently. B1s are younger than L1s, having arisen just before the divergence of the common ancestor of rodents, ~ 65 MYA [34], and they are specific to rodents. Other SINEs, including B2, B4 and ID elements, are also present in rodent genomes [19]. SINE families have been interacting with L1s for more than 100 MYA, and fossil remnants of extinct SINE families are detectable in well-characterized mammalian genomes [18, 35]. Despite being targeted by a slew of host restriction mechanisms, L1 and B1 compose approximately a quarter of a typical rodent genome [4, 7]. For example, in the mouse genome, there are ~ 599,000 total copies of L1, responsible for ~ 19% of the genome [4], of which ~ 3000 copies are potentially functional [36], and ~ 564,000 copies of B1, responsible for ~ 3% of the genome [4].

LINEs and SINEs have considerable impact on the mammalian genome, although they were traditionally viewed as “junk DNA”. As LINEs and SINEs, including L1s and B1s, retrotranspose and recombine, they introduce genome instability [37] and cause disease [38]. These elements may occasionally be co-opted by the host to serve host certain functions, such as their proposed roles in neuro-plasticity [39, 40], X chromosome inactivation [41, 42], regulatory functions [43, 44], and DNA break-repair [45]. Our current picture of the effects of retrotransposition does not take into account what effect silencing of LINEs and SINEs might have on these proposed functions.

Since L1 retrotransposition is under strict control by multiple host defenses [46], it might seem reasonable for the host to occasionally win the evolutionary arms race with L1s, resulting in loss of L1 activity (L1 extinction). Yet, L1 extinctions are relatively rare. Factors contributing to the rarity of L1 extinction could be but not limited to co-option of L1s for certain essential function to the host. Mammalian L1s are not known to move horizontally, although ancient L1s might have been able to do so given evidence of other non-LTR retrotransposons [47]. The unlikely replacement of L1s by horizontal movement would be evident as a mismatch between L1 phylogeny and host phylogeny. Therefore, mammalian L1 extinctions would affect all derived host species. Two factors are of note here. First, clades with early L1 extinctions could have given rise to large mammalian lineages without L1 activity and be easily detected because of both the number of species affected and the deterioration of the remnant sequences in the genome. Secondly, recent extinctions will be difficult to differentiate from periods of L1 quiescence. To clarify the terms related to loss of L1 activity in this work, we refer to a period of low L1 activity as “quiescence” and complete loss of L1 activity as “extinction”. Given the large phylogenetic impact of early extinctions, one might expect L1s to eventually become extinct in most mammalian genomes, and yet L1s have persisted throughout the entire evolutionary history of their placental mammal and marsupial hosts. Thus, either most L1 extinctions are either recent or rare, or mammalian lineages subject to ancient L1 extinctions do not persist or they give rise to few new species. Understanding the dynamics of L1 extinction will be as important as understanding the dynamics of L1 activity in sorting out the impact of L1s on mammalian genome evolution.

**Fig. 1:**
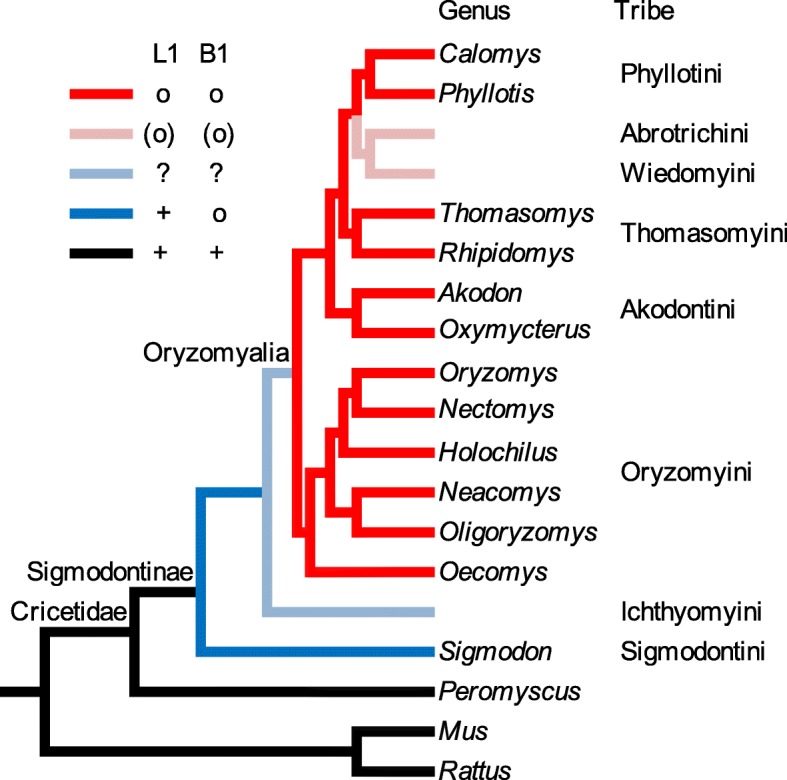
Phylogeny of the sigmodontine rodents. L1 and B1 activity displayed on the phylogeny shows that L1 extinction preceded that of B1. The phylogeny is based on Schenk et al. [57]. Taxa are the genera sampled; tribes are indicated on the right side of the taxa. Eight of the nine tribes and 12 of the 14 genera sampled by Rinehart et al. [52] are shown. L1 and B1 activity of each taxon is demonstrated by color: black, active L1 and B1; deep blue, active L1 and inactive B1; deep red, inactive L1 and inactive B1; light blue, L1 activity cannot be inferred; and, light red, L1 and B1 can be inferred to be inactive. “+” corresponds to active L1 and B1 and “o” corresponds to inactive L1 and B1

Several cases of L1 extinction have been proposed in the literature [48–56] and two of these are deep extinction events that cover major groups of mammals [50–52]. One of the major L1 extinctions occurred in a large group of South American rodents and includes most species in Sigmodontinae. Sigmodontinae is a subfamily of the Cricetidae family, including approximately 377 species classified into 74 genera in nine tribes (Fig. 1) [58] and thus contains 7–8% of the estimated 5000 mammalian species [59]. Given that B1 retrotransposition is dependent on that of L1, it is expected that B1s should lose their activity simultaneously with L1s. However, the B1 extinction in Sigmodontinae appears to have preceded that of L1s based on samples from 14 genera in five tribes [50–52], where the basal genus *Sigmodon* carries inactive B1 and active L1, and the descendant genera carry both inactive L1 and B1 (Fig. 1). It has also been shown that loss of L1 and B1 activity follows the expansion of a group of endogenous retroviruses [60, 61].

It was previously hypothesized that L1 can experience long-term quiescence as a “stealth driver” [62], and that B1 extinction could happen during this period of L1 quiescence [52]. Since B1s are more prone to mutations than the average sequence due to enriched CpG content [24], Rinehart et al. [52] hypothesized that B1 was unable to retrotranspose at a high enough rate during L1 quiescence to replace their active copies, accumulating debilitating mutations more rapidly than L1s. When a more active family of L1 emerged in the Sigmodontini, it was hypothesized that B1 was too degraded to retrotranspose, resulting in B1 extinction even in the presence of high L1 activity.

In this study, we investigate the evolution histories of L1 and B1 spanning the time of their extinctions and the radiation of the extant species in Sigmodontinae (Fig. 1). Since the group carrying extinct L1s and B1s (Oryzomyalia, Fig. 1) shares a common ancestor, we used the marsh rice rat *Oryzomys palustris* to represent this group, hereafter referred to as the “L1-extinct clade”. We used the hispid cotton rat *Sigmodon hispidus* to represent the clade carrying active L1 but inactive B1, hereafter referred to as the “basal group”. We used the deer mouse *Peromyscus maniculatus* to represent a closely related clade carrying both active L1 and B1, hereafter referred to as the “outgroup”.

Using unassembled genome sequences from the species representing the L1-extinct clade and the basal group, we show that the activity of L1 and B1 families preceeding the divergence of the clades is comparable in the current genomes of the two groups. L1 families had been steadily replaced before the split of the two groups and maintained activity after the split of the basal group and the L1-extinct clade. Shortly after this split L1 activity ceased in the L1-extinct clade but became highly active in the basal group. B1s, on the other hand, had a very large increase in activity prior to the split between the L1-extinct clade and the basal group, and there is no evidence of activity in the two groups following their divergence.

## Results

To investigate the history of L1 retrotransposition in *O. palustris* and *S. hispidus*, we used COSEG [63] to identify closely related L1 groups based on shared, co-segregating sites as described in Methods. We follow the convention of COSEG to designate these groups as *subfamilies*. RepeatMasker [63] was used to assign genomic L1 copies to COSEG-generated subfamilies, and seven subfamilies with no assigned sequences were removed from further consideration, leaving 47 subfamilies for further analysis.

To examine the activity of L1s in *O. palustris* and *S. hispidus*, we searched the trace files of both genomes separately with the consensus sequences of the abovementioned 47 subfamilies and identified 19,254 sequences in *O. palustris* and 90,526 in *S. hispidus*. The relative age of each sequence was approximated by its percent divergence from the corresponding subfamily consensus - the higher the percent divergence, the older the sequence. The peak of the distribution was used as an approximation of the divergence of the subfamily (Additional file 3 Table S1). Given the possible changes of evolution rate in the detectable range of L1 evolutionary history, a global conversion from percent divergence to time is challenging. However, because of the shared evolutionary history of *O. palustris* and *S. hispidus*, we are able to use percent divergence as a reasonably good marker to compare the relative ages of L1 subfamilies of the two species, as is typical in studies of transposable elements [63].

**Fig. 2:**
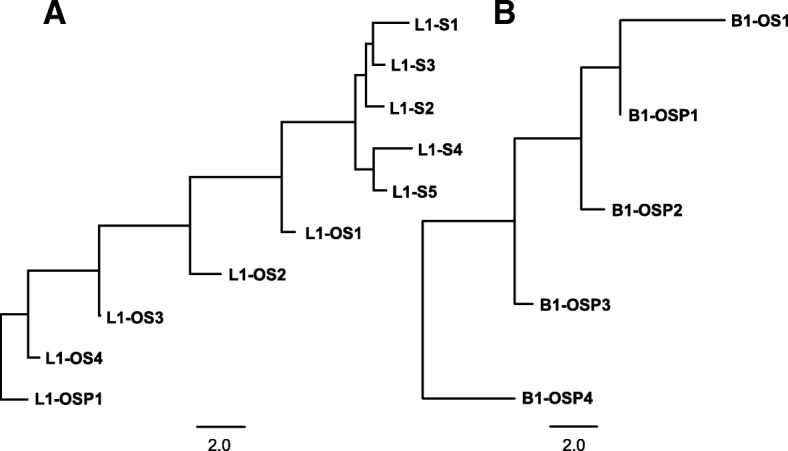
Phylogenies of L1 and B1 families. The phylogenies show species specificity of L1 and B1 families, and that *S. hispidus*-specific L1 families exist, but not B1 families. Panel **a** shows the L1 tree and **b** shows the B1 tree. To reflect divergence of the families, the trees were based on the distances between them. The distance between any two families was calculated by taking the average pairwise distance of the consensus sequences of subfamilies that belong to each family. Numbering of the L1 and B1 families is in chronological order of each element - similarly numbered L1 and B1 families do not necessarily correspond to the same time period

**Fig. 3:**
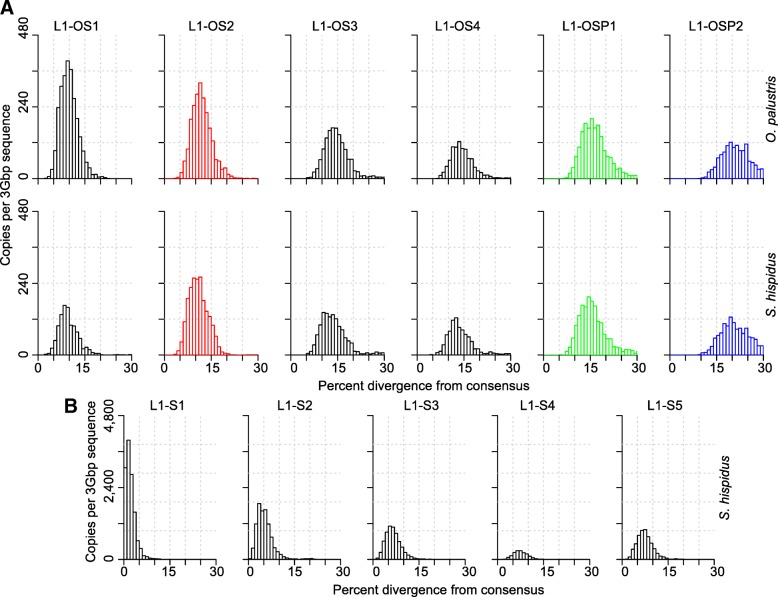
Divergence distribution of L1 families. L1 families were shared and had similar copy numbers in *O. palustris* and *S. hispidus* until just prior to the extinction of L1. L1 experienced a slow-down (middle left panel) in *S. hispidus* until it recovers (bottom panels - note that the copy number axis is 10x that of the top and middle panels). L1 families in each row are arranged in chronological order with the youngest families on the left. The species analyzed in each row is indicated at the right. Names of families are noted on the top of each panel. L1 copy number is plotted by percent divergence from the corresponding subfamily consensus in 1% bins. The divergence of each family is approximated by the peak of the distribution. L1 copy numbers are normalized as copies per three Gbp of MiSeq sequence which approximates the copy number per haploid genome. Panel **a** shows the shared families and panel **b** shows the *Sigmodon*-specific families. L1 and B1 families (in all figures) that correspond to similar divergence are given the same color and this color coding is consistent in all figures showing L1 and B1 divergence: L1-OS2, red; L1-OSP1, green; and, L1-OSP2, blue

Subfamily consensus sequences were also used to infer phylogenetic relationships between subfamilies (Additional file 1: Figure S1). Subsequently, phylogenetic relationships and sequence similarities between subfamilies were used to assign subfamilies to families with the stipulation that the pairwise distance between subfamilies within a family be no greater than 3.5%. This distance was determined operationally based on the divergences among phylogenetically clustered subfamilies. Clusters of subfamilies that were similar at the sequence level but differed in divergence were assigned to different families. This process identified five families specific to *S. hispidus* (L1-S1 to L1-S5), four families shared by *O. palustris* and *S. hispidus* (L1-OS1 to L1-OS4) and two shared by *P. maniculatus*, *O. palustris* and *S. hispidus* (L1-OSP1 and L1-OSP2, Additional file 3: Table S1). A distance-based phylogeny reflecting the relationship between L1 families is presented in Fig. 2a. Individual sequences were assigned to the families to which their subfamilies belong; the divergence within a family is based on the distance of each sequence from its subfamily consensus (Fig. 3).

As expected, sequences from L1 families shared by *O. palustris* and *S. hispidus* are present in both genomes, and these shared families are fairly synchronized in time and comparable in copy number (Fig. 3a). L1-OS1 is the only shared L1 family between *O. palustris* and *S. hispidus* that shows a difference: it is the youngest shared L1 family, the last active L1 family prior to the L1 extinction, and has ~ 1.5-fold higher copy numbers per Gbp of sequence in *O. palustris* than in *S. hispidus*. This difference in L1-OS1 deposition between *O. palustris* and *S. hispidus* suggests that L1s remained active in the L1-extinct clade after the separation of that group from the basal group. The *Sigmodon*-specific L1 families (Fig. 3b, families S1–5) experienced substantial amplification after divergence from the L1-extinct clade, whereas no *Oryzomys*-specific subfamilies were identified by COSEG. The *Sigmodon*-specific subfamilies had a few sequences from the *O. palustris* genome assigned to them, but these assignments appear to be anomalous since the sequences are highly divergent from the subfamily consensus sequences (Additional file 3: Table S1). Thus, L1 experienced an expansion (L1-OS1) in the lineage leading to Oryzomyalia immediately before L1 extinction, while the lineage leading to Sigmodontini experienced a delayed but much larger L1 expansion.

**Fig. 4:**
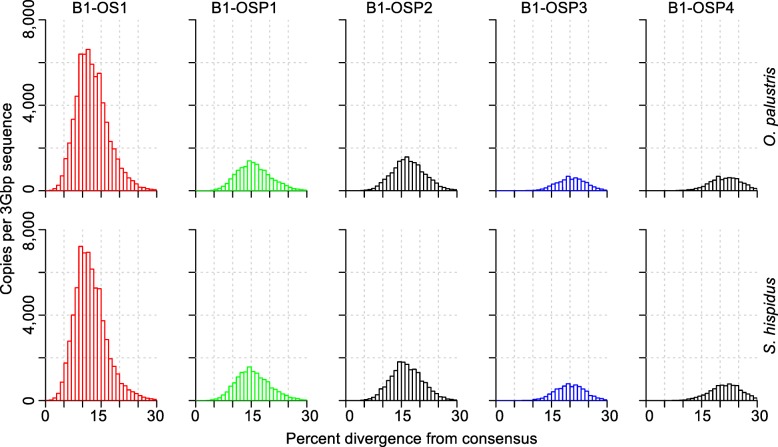
Divergence distribution of B1 families. B1 families in each row are arranged in chronological order with the youngest families on the left. The species analyzed in each row is indicated at the right. Names of families are noted on the top of each panel. B1 copy number is plotted by percent divergence from the corresponding subfamily consensus in 1% windows. The divergence of each family is approximated by the peak of the distribution. B1 copy numbers are normalized as copies per three Gbp of MiSeq sequence which approximates the copy number per haploid genome. L1 and B1 families (in all figures) that correspond to similar divergence are given the same color and this color coding is consistent in all figures showing L1 and B1 divergence: B1-OS1, red; B1-OSP1, green; and, B1-OSP3, blue

In order to study the B1 dynamics in sigmodontine rodents, we performed the analysis on B1 similarly to that done on L1. Because of the short length and CpG-rich nature of B1, we required twice as many sequences to form a subfamily in the second round of COSEG as described in Methods. The analysis revealed 30 subfamilies and five families of B1 in both species (Additional file 4: Table S2). A distance-based phylogeny reflecting the relationships between B1 families is presented in Fig. 2b. One of the families (B1-OS1) is shared by *O. palustris* and *S. hispidus* and the subfamilies within B1-OS1 form a polytomy on the distance-based phylogeny (data not shown). The other four B1 families (B1-OSP1–4) are shared by *O. palustris*, *S. hispidus* and *P. maniculatus*. The representation of these families in both *O. palustris* and *S. hispidus* genomes is fairly synchronized in time and comparable in copy number (Fig. 4). Since the outgroup, represented by *P. maniculatus*, carries both active L1s and B1s, we know that B1 extinction happened after the split of the outgroup, yet the point at which B1 lost activity in the basal group is to be determined. Here we show that the peak of the most recent B1 family resides at ~ 11.1% divergence in *O. palustris* and ~ 10.7% in *S. hispidus* (Additional file 4: Table S2). These peaks reside in the same divergence window as L1-OS2 (~ 11.1% in *O. palustris* and ~ 10.3% in *S. hispidus*, Additional file 3: Table S1), suggesting that B1-OS1 is coincident in time with L1-OS2. Exclusion of CpG sites when calculating percent divergence reduces the variation between the L1 and B1 clocks, but we acknowledge that other minor mutation rate variations between L1s and B1s might still exist. Since L1-OS2 is the youngest L1 family prior to the separation of the basal group and the L1-extinct clade, the last wave of B1 retrotransposition likely preceded the extinction of L1.

## Discussion

In this paper we explore the tempo of L1 and B1 activity surrounding the extinction of both elements that occurred in most species within the rodent subfamily Sigmodontinae. This work is made possible by sequencing methods that allow us to gather large amounts of sequence data and by the availability of a robust species phylogeny for the group (Fig. 1). A recent phylogenetic analysis of muroid rodents [57] indicates that the tribe Sigmodontini is basal in the Sigmodontinae and sister to the tribe Ichthyomyini. These two tribes are sister to a large, polytomic group (the Oryzomyalia) which includes the remaining six tribes. The subfamily is the result of a rapid radiation of rodents into South America about 5 MYA [64]. Previous work indicated that L1s are extinct in the Oryzomyalia but active in the Sigmodontini, which is composed of 14 species in one genus, *Sigmodon*. A summary of total L1s and B1s in *S. hispidus* and *O. palustris* (Additional file 2: Figure S2) agrees with this pattern of extinction. L1 extinction in the Oryzomyalia has been documented in 13 genera distributed across four tribes spanning this group (Fig. 1). Evidence for this L1 extinction included sequence divergence between L1s cloned by a method that enriches for recently transposed elements [65], and faint hybridization along with the absence of species- or genus-specific bands in a Southern blot of the 13 genera when probed with L1 [51].

**Fig. 5:**
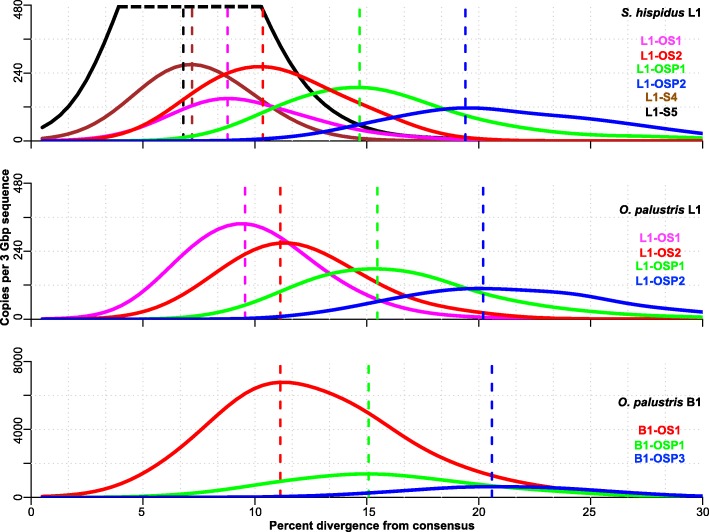
Comparison of the divergence and copy numbers of L1 and B1 families. Curves represent the divergence distribution of L1 and B1 families based on kernel smoothing of the 1% divergence bin size histogram of the family. Vertical dashed lines represent the peaks of the distributions. L1 and B1 families (in all figures) that correspond to similar divergence are given the same color and this color coding is consistent in all figures showing L1 and B1 divergence: L1-OS2 and B1-OS1, red; L1-OSP1 and B1-OSP1, green; and, L1-OSP2 and B1-OSP3, blue. The oldest two *S. hispidus*-specific L1 families are also shown: L1-S5, black; L1-S4, brown. The top portion of the L1-S5 curve (above the horizontal black dashed line) is truncated for visualization purposes, but its peak coordinate is shown by the vertical black dashed line

We reconstructed the shared evolutionary history of L1s and B1s in Sigmodontinae in the period preceding and following extinction of these elements. Our results suggest that L1 master elements were replaced steadily prior to the extinction of both L1 and B1. This is reflected by the consecutive series of L1 families shared by *O. palustris* and *S. hispidus* after their divergence from *Peromyscus*. B1 elements did not appear to take advantage of every wave of L1 activity, but a wave of L1 retrotransposition (family L1-OS2, red color in Figs. 3 and 5) corresponds to the B1 retrotransposition peak just prior to B1 extinction (B1-OS1, red color in Figs. 4 and 5).

By determining the minimum distance of individual L1s and B1s from their consensuses in species spanning 12 genera of the Sigmodontinae, Rinehart et al. [52] proposed that L1 extinction occurred after the split between the L1-extinct clade and *Sigmodon*, the basal genus. We further refine this timeframe in a summary diagram showing the higher level of L1-OS1 activity in *O. palustris* compared to *S. hispidus* (Fig. 5, magenta color). This supports the conclusion that there was some level of L1 activity in both species after the split, and that the events leading to L1 extinction also happened after the split, rather than via recovery of L1 activity in *S. hispidus* as previously suggested [51]. The evolutionary history of B1 in *O. palustris* and *S. hispidus* is comparable. New B1 deposition into the genome was unremarkable except for the large burst in both species in the period directly preceding B1 extinction (B1-OS1, red color in Figs. 4 and 5). Given the short length of B1s, it is more difficult to identify subfamily clusters, so our estimation of the timing of B1 extinction is weaker than for L1. However, two lines of evidence suggest that the last burst of B1 activity occurred prior to the split between the L1-extinct and basal groups. Firstly, the peak activity of B1-OS1 corresponds most closely to the peak activity of L1-OS2, which appears to precede the split of these two rodent clades (Fig. 5, red color). Secondly, there is no indication of difference in activity for any of the B1 subfamilies in *O. palustris* and *S. hispidus* (Additional file 4: Table S2), as was the case for L1 (Additional file 3: Table S1).

Estimation of retrotransposition rate based on historical L1 copy numbers could be affected by the excision rate of the host genome or detection limit of the algorithm. Although no known mechanism specifically targets L1 and B1 for excision, mammalian genomes have been constantly expelling sequences by various mechanisms and the excision rate varies in different groups [66]. Older insertions are exposed to the non-targeted excision mechanisms for longer time, thus fewer copies of the older families are expected to be represented in the genome. Old L1 and B1 copies also suffer from more limited recognition by the available algorithms. The sequences detectable by RepeatMasker decrease drastically beyond 30% divergence. Since the mutation rate in the rodent lineage is one of the highest in all mammals, 30% divergence in L1 and B1 only traces back to the common ancestor of sigmodontine rodents and *P. maniculatus*, while similar studies on bats [49] and primates [67, 68] trace back to the common ancestor of mammals. Fortunately, *P. maniculatus* carries both active L1s and B1s and is close enough to serve as an outgroup in this study. We were able to identify two L1 families shared by *O. palustris*, *S. hispidus* and *P. maniculatus*, L1-OSP1 and L1-OSP2. However, there is an advantage of studying rodents in this type of evolutionary study. Since the mutation rate in the rodent lineage is higher than that of other mammals, evolution of L1 and B1 subfamilies over a given period of time will show greater divergence compared to more slowly evolving species. This gives the divergence distributions of L1s and B1s higher resolution and allows us to discern subtle differences between subfamily divergence.

## Conclusions

The patterns of historical L1 and B1 activity reveal the critical time frame at which retrotransposition rates diverged in the ancestral hosts. It is apparent that L1s were still active at the time when the ancestral lineages of Oryzomyalia and Sigmodontinae split. Thus, rather than quiescence of L1 in the common ancestor with resurgence in the sigmodontine lineage, the extinction of L1 appears to have occurred after the split and only in the Oryzomyine lineage. L1s are extinct in Oryzomyalia but active in Sigmodontini. B1s are extinct in Oryzomyalia and Sigmodontini. However, the status of both L1s and B1s in the intermediate tribe, Ichthyomyini, is unknown. Thus, L1 extinction from this single event likely affects between 345 and 362 species, or about 7% of all mammalian species.

Our study also reveals the largest and final wave of B1 expansion indeed occurred in the common ancestor of the host lineages. This huge burst of B1 activity suggests an explanation other than L1 quiescence, and the subsequent deficit of L1 proteins, for its extinction. Given that transposable elements can be involved in an evolutionary arms race with host restriction factors [69], it is possible that the radical expansion of B1 triggered stronger host defense and eventually led to the extinction of L1 and B1. Therefore, reconstruction of the evolutionary history of L1 and B1 host restriction factors in relevant rodent species could be the key to revealing the mechanism of L1 and B1 extinction.

## Methods

*O. palustris* DNA was obtained from the Natural Sciences Research Laboratory in The Museum of Texas Tech University (tissue ID: TK28621), and *S. hispidus* DNA was obtained from Texas Cooperative Wildlife Collection of the Texas A&M University (tissue ID: MUR15). Whole genome sequencing of each species was done in separate batches using MiSeq (Illumina, Inc., San Diego, CA) at the IBEST Genomic Resources Core (University of Idaho, Moscow, ID). Paired-end libraries were generated with an insert size of 450–550 bp; ~ 13 and 14 million total reads were generated for *O. palustris* and *S. hispidus*, respectively. Sequences were processed with SeqyClean (https://bitbucket.org/izhbannikov/seqyclean) and the paired-ends were joined with FLASH [70]. Genome coverage was equivalent to approximately 1.5X; 5.47 Gbp of sequence were generated for *O. palustris* and 6.06 Gbp for *S. hispidus*, but we note that genome size within the sigmodontine rodents varies. Although the genome size of *O. palustris* is not documented to our knowledge, the genome size of sister species in *Oryzomys* suggest that *Sigmodon* genomes are 11–16% larger than those of *Oryzomys* [71].

L1 reconstructions for both species were generated based on partial genomic sequences generated by 454 Pyrosequencing (Roche Applied Science, Penzberg, Germany) at the IBEST Genomic Resources Core, 203 Mbp of sequence for *O. palustris* and 214 Mbp for *S. hispidus*. *P. maniculatus* genome trace files were obtained from NCBI FTP site (ftp://ftp.ncbi.nlm.nih.gov/pub/TraceDB/peromyscus_maniculatus/). Reconstruction of the 3′ ends of *O. palustris* and *S. hispidus* L1s started with a 575 bp consensus seed in the 3′ half of L1 ORF2 generated following Cantrell et al. [65]. A bioinformatic pipeline for reconstructing a full length L1 is described by Yang et al. [49]. Briefly, sequences were acquired from the genome trace files based on percent identity [72, 73]. The overhangs of the found sequences allowed the creation of new seeds at both ends of the L1 fragment and were used to initiate another round of query. In this case, the reconstruction walk was repeated in the 3′ direction until the 3′ end of ORF2 was reached. Percent identity cutoff was set at 92% for *O. palustris* to capture the most recently active sequences; a higher percent identity (97–99%) and overhang length of at least 100 bp were used for *S. hispidus*. This assured a satisfactory consensus and exclusion of older L1 elements for each species. The 3′ 300 bp of the reconstructed L1s were then used as the reference sequences for COSEG analysis described below.

B1 sequences from Rinehart et al. [52] were used as starting seeds for B1 analysis. The PCR-amplified B1s from *O. palustris* and *S. hispidus* were aligned with Lasergene MegAlign (DNASTAR, Madison, WI) and the consensus sequence (146 bp) was used as the reference sequence for COSEG analysis.

L1 and B1 subfamilies in *O. palustris* and *S. hispidus* were identified and characterized in similar fashion as described below and are summarized in Additional file 3: Table S1 and Additional file 4: Table S2. The alignment of L1 and B1 subfamily sequences are available in Additional file 5 and Additional file 6.

The reconstructed 300 bp sequences from the 3′ end of *O. palustris* and *S. hispidus* L1 ORF2 were each used as the initial L1 query sequences, and the full length B1 consensuses from each species, based on Rinehart et al. [52], were used as the initial B1 query sequences. *O. palustris* and *S. hispidus* MiSeq genomic DNA libraries (processed reads without assembly) were queried using RepeatMasker [63] with default parameters. This initial step was conducted to identify any sequence that is potentially homologous to L1/B1, which saves computation time of the following steps by avoiding the need to query all sequencing reads directly. Hits from RepeatMasker searches were filtered for > 90% coverage of the query sequence and subsequently used for the first COSEG [63] (http://www.repeatmasker.org/COSEGDownload.html) run to identify subfamilies based on shared, co-segregating sequence variants. All COSEG runs were conducted under default parameters except as noted. Parameters were set such that at least 250 sequences were required to form a L1 subfamily and 1000 were required to form a B1 subfamily. In order to identify older subfamilies, the consensus sequences of the subfamilies identified by the first COSEG run were used as queries to again search the *O. palustris* and *S. hispidus* MiSeq libraries using RepeatMasker. The identified sequences from the second RepeatMasker run were filtered for > 90% coverage and extracted. *O. palustris* and *S. hispidus* sequences are combined and a second COSEG run was carried out on the combined sequences. To avoid the possible formation of random subfamilies due to the short length of B1 and the high copy number of the detected sequences, the sequences required to form a subfamily was increased from 1000 (for the former separate run) to 2000, whereas this number for L1 remained unchanged at 250. The consensus sequences of the resulting COSEG subfamilies were trimmed to exclude ends that were not common to all subfamilies and the CpG sites were removed and, thus, treated as gaps by RepeatMasker and not counted for the divergence calculation. These modified subfamily consensus sequences were used for a final query of the individual *O. palustris* and *S. hispidus* MiSeq libraries using RepeatMasker. Sequences from this third run were assigned to subfamilies based on percent divergence and this information was stored for further analysis.

*P. maniculatus* genome trace files were data-mined in a similar fashion through a single round of RepeatMasker and COSEG. The *O. palustris* L1 and B1 sequences described above were used as the initial query seeds for this run. Selected *P. maniculatus* subfamilies were used to demarcate the divergence of the subfamilies identified in the *O. palustris* and *S. hispidus* genomes (Fig. 3).

Subfamily consensus sequences generated by the second COSEG run of the *O. palustris* and *S. hispidus* libraries were combined and aligned with MegAlign using the Clustal W method for L1 or Clustal V method for B1 and a distance matrix was calculated based on the alignment. Due to the use of subfamily consensuses, gaps are rare in the alignment and hence kept. CpG sites were manually removed from the alignments. Based on the alignments, maximum likelihood trees were constructed using PhyML [74] with the GTR + I + G model and 100 bootstrap replicates (Additional file 1: Figure S1). L1 and B1 sequences were then assigned to families based on the topology of the tree and a no more than 3.5% within-family pairwise distance from their subfamily consensuses for L1 and 4.4% for B1. Given that the L1 and B1 masters are constantly being replaced during evolution, perfect designation of large families is not possible. The 3.5% threshold was chosen so as to cluster closely related subfamilies without inflating the number of families. Families are named according to their species-specificity and divergence: “S” indicates *Sigmodon*-specific families, “OS” for families shared by *Sigmodon* and *Oryzomys* and “OSP” for families shared by *Sigmodon*, *Oryzomys* and *Peromyscus*; numbers in family names indicates the divergence of a family within the family group with “1” being the youngest. Family consensus sequences were generated in MegAlign (DNAStar, Madison, WI) using the consensus sequences of subfamilies belonging to each family. Alignment and phylogeny of families were generated as described above for subfamilies. Histograms of L1 and B1 divergence distributions were generated by R [75] histogram function using a bin size of 1% (Figs. 3 and 4). Percent divergence corresponding to retrotransposition peaks of individual families and subfamilies were determined by R using the kernel smoothing function with 0.4% bandwidth (Additional file 3: Table S1 and Additional file 4: Table S2).

To avoid any bias introduced by using the consensus-based seeds, we performed a similar analysis on the L1s in *S. hispidus* using RepeatScout [76], which is a de novo repeat identification method that does not use any priori of known repeats. RepeatScout was run with default parameters on the processed MiSeq library of *S. hispidus* to find repetitive sequences. Identified repeats were then annotated using RepeatMasker. All L1-like sequences that overlaps with the 3′ 300 bp of *S. hispidus* L1 ORF2 were used as seeds to perform a COSEG analysis of L1s in *S. hispidus* following the approach described above. All RepeatScout-based L1 subfamilies (Additional file 7) in *S. hispidus* were within 3.5% divergence of a COSEG-defined subfamily as described above, and hence can be assigned to the same L1 family.

## Additional files

Additional file 1: **Figure S1.** Maximum likelihood phylogeny of detected L1 subfamilies. Reconstructed *O. palustris* and *S. hispidus* L1s, labeled ‘seed’, and *P. maniculatus* subfamilies 5 and 6 are included as markers. The tree was constructed using PhyML [74] with the GTR + I + G model and 100 bootstrap replicates. Bootstrap values > 80% are shown. (PDF 39 kb)

Additional file 2: **Figure S2.** Divergence distribution of all detected L1 and B1 sequences. Percent divergence from the corresponding subfamily consensus sequences are plotted in 1% bins. Species and retrotransposon names are indicated at the top of each panel. (PDF 36 kb)

Additional file 3: **Table S1.** Statistics and designation of L1 subfamilies and families. “Ory” stands for *O. palustris* and “Sig” stands for *S. hispidus*. “Peak” indicates the peak of the L1 divergence distribution of the subfamily or family identified by kernel smoothing. Copy numbers are normalized as copies per three Gbp of MiSeq sequence used for the search, which approximates the copy number per haploid genome. Designation of families is only shown after the first subfamily that belongs to it; all subsequent subfamilies belong to this family until the demarcation of the next family. Characters in family names: “S” represents *S. hispidus*-specific, “OS” for shared by *O. palustris* and *S. hispidus* and “OSP” for shared by *O. palustris*, *S. hispidus* and *P. maniculatus*. Numbers in the family names reflect their divergence among the family group with “1” being the youngest. Copy numbers of families are rounded sums of subfamily copy numbers per three Gbp of sequences and, thus, are occasionally off by one. (XLSX 14 kb)

Additional file 4: **Table S2.** Statistics and designation of B1 subfamilies and families. “Ory” stands for *O. palustris* and “Sig” stands for *S. hispidus*. “Peak” indicates the peak of the B1 divergence distribution of the subfamily or family identified by kernel smoothing. Copy numbers are normalized as copies per three Gbp of MiSeq sequence used for the search. Designation of families is only shown after the first subfamily that belongs to it; all subsequent subfamilies belong to this family until the demarcation of the next family. Characters in family names: “OS” represents families shared by *O. palustris* and *S. hispidus* and “OSP” for families shared by *O. palustris*, *S. hispidus* and *P. maniculatus*. Numbers in the family names reflect their divergence within the family group with “1” being the youngest. Copy numbers of families are rounded sums of subfamily copy numbers per three Gbp of sequences.and *S. hispidus* genome. CpG sites with evidence of methylation-induced mutations were removed from the alignment. (XLSX 13 kb)

Additional file 5: Alignment of L1 subfamily sequences used in the tables and figures. CpG sites with evidence of methylation-induced mutations were removed from the alignment. (FA 15 kb)

Additional file 6: Alignment of B1 subfamily sequences used in the tables and figures. CpG sites with evidence of methylation-induced mutations were removed from the alignment. (FA 7 kb)

Additional file 7: Alignment of L1 subfamily sequences based on RepeatScout-generated seeds and *S. hispidus* genome. CpG sites with evidence of methylation-induced mutations were removed from the alignment. (FA 21 kb)

## Abbreviations

LINE: Long INterspersed Element
MYA: Million Years Ago
*O. palustris*: *Oryzomys palustris*
ORF: Open Reading Frame
*P. maniculatus*: *Peromyscus maniculatus*
*S. hispidus*: *Sigmodon hispidus*
SINE: Short INterspersed Element
