# Supplementary figures and images for: Tracing the history of LINE and SINE extinction in sigmodontine rodents
Source: Mob DNA. 2019 May 21;10:22. doi: 10.1186/s13100-019-0164-5 (PMC6530004; doi:10.1186/s13100-019-0164-5)

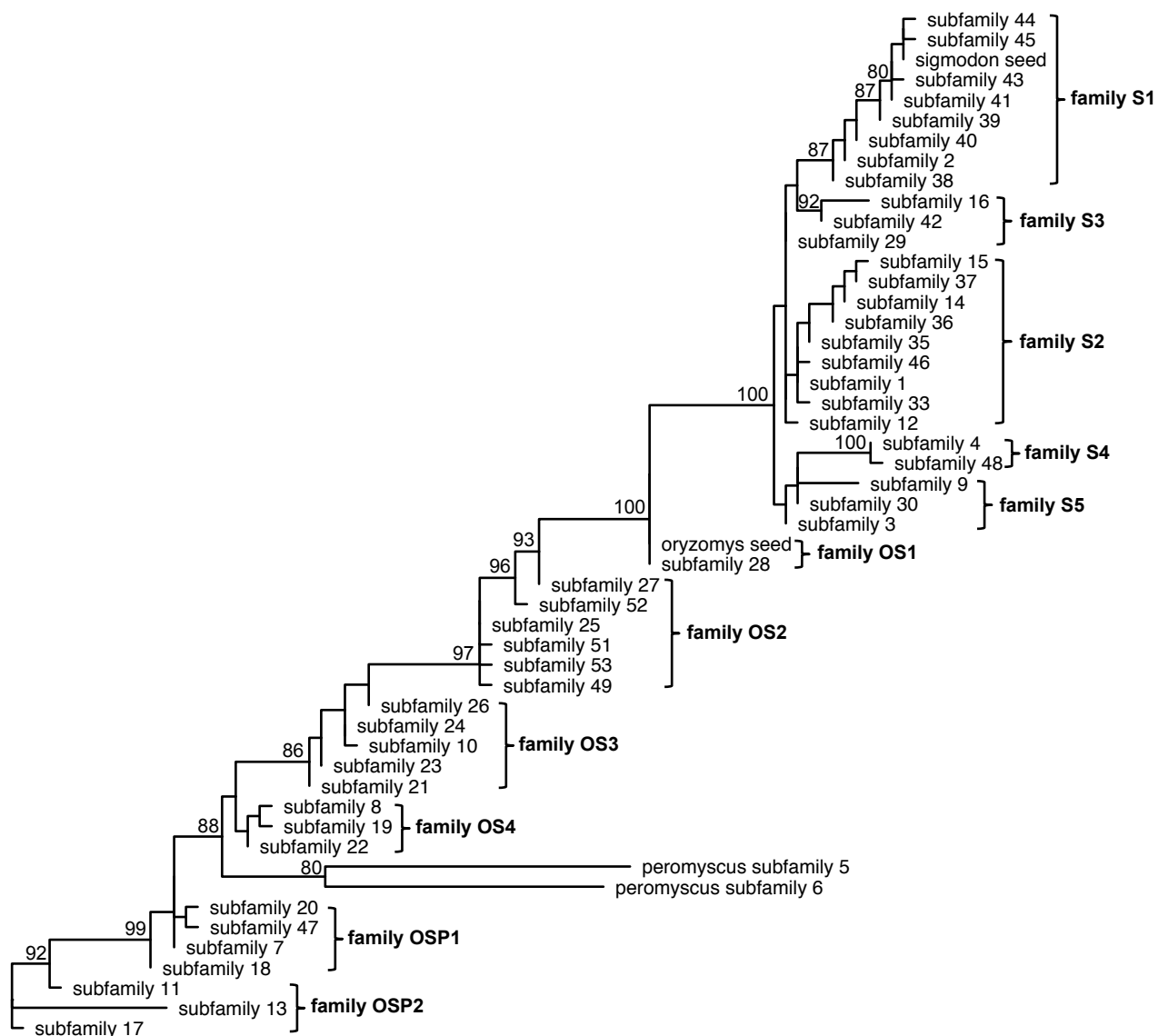

Supplement: Supplementary file 1 — Figure S1. Maximum likelihood phylogeny of detected L1 subfamilies. Reconstructed O. palustris and S. hispidus L1s, labeled ‘seed’, and P. maniculatus subfamilies 5 and 6 are included as markers. The tree was constructed using PhyML [74] with the GTR + I + G model and 100 bootstrap replicates. Bootstrap values > 80% are shown. (PDF 39 kb) [file 13100_2019_164_MOESM1_ESM.pdf]

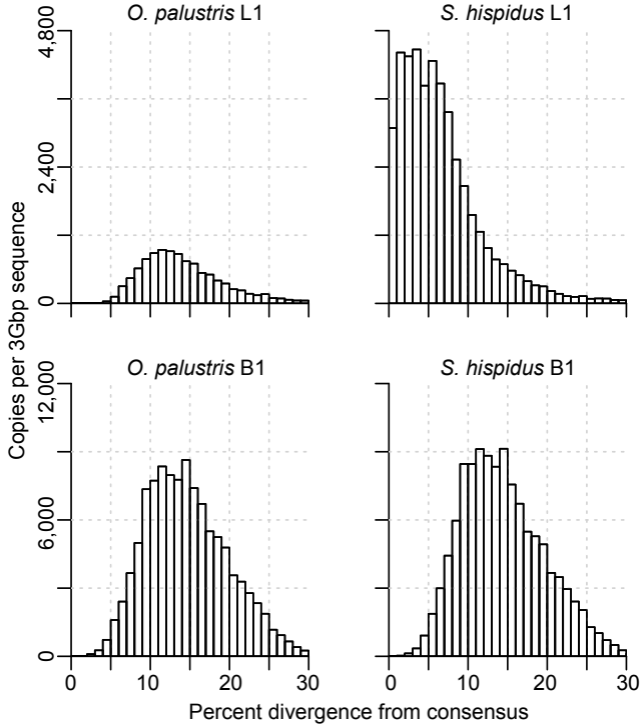

Supplement: Supplementary file 2 — Figure S2. Divergence distribution of all detected L1 and B1 sequences. Percent divergence from the corresponding subfamily consensus sequences are plotted in 1% bins. Species and retrotransposon names are indicated at the top of each panel. (PDF 36 kb) [file 13100_2019_164_MOESM2_ESM.pdf]
